# Top-Down Identification and Sequence Analysis of Small Membrane Proteins Using MALDI-MS/MS

**DOI:** 10.1021/jasms.2c00102

**Published:** 2022-06-27

**Authors:** Jakob Meier-Credo, Laura Preiss, Imke Wüllenweber, Anja Resemann, Christoph Nordmann, Jure Zabret, Detlev Suckau, Hartmut Michel, Marc M. Nowaczyk, Thomas Meier, Julian D. Langer

**Affiliations:** † Proteomics, Max Planck Institute of Biophysics, Max-von-Laue-Strasse 3, 60438 Frankfurt am Main, Germany; ‡ Proteomics, Max Planck Institute for Brain Research, Max-von-Laue-Strasse 4, 60438 Frankfurt am Main, Germany; § Structural Biology, Max Planck Institute of Biophysics, Max-von-Laue-Strasse 3, 60438 Frankfurt am Main, Germany; ∥ Department of Life Sciences, Imperial College London, Exhibition Road, SW7 2AZ London, United Kingdom; ⊥ Bruker Daltonics GmbH & Co. KG, Fahrenheitstrasse 4, 28359 Bremen, Germany; # Department of Plant Biochemistry, Ruhr University Bochum, 44780 Bochum, Germany; 7 Molecular Membrane Biology, Max Planck Institute of Biophysics, Max-von-Laue-Strasse 3, 60438 Frankfurt am Main, Germany

## Abstract

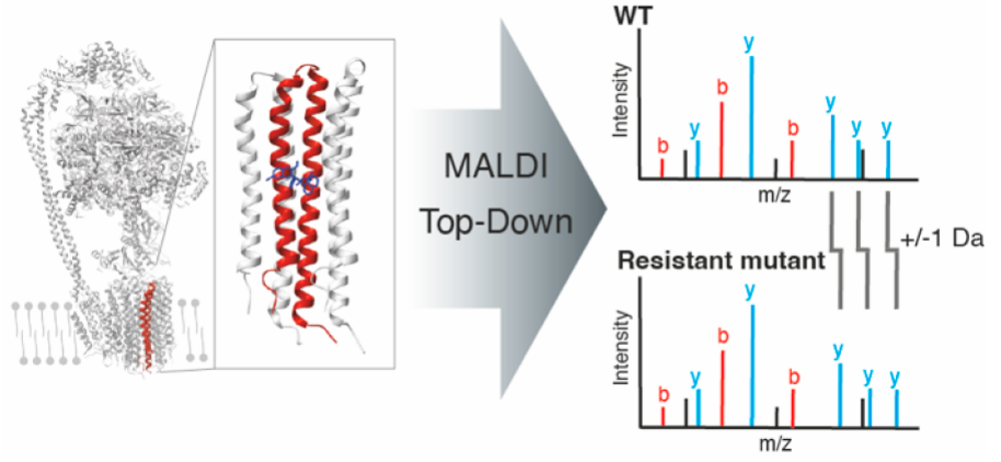

Identification and sequence determination by mass spectrometry have become routine analyses for soluble proteins. Membrane proteins, however, remain challenging targets due to their hydrophobicity and poor annotation. In particular small membrane proteins often remain unnoticed as they are largely inaccessible to Bottom-Up proteomics. Recent advances in structural biology, though, have led to multiple membrane protein complex structures being determined at sufficiently high resolution to detect uncharacterized, small subunits. In this work we offer a guide for the mass spectrometric characterization of solvent extraction-based purifications of small membrane proteins isolated from protein complexes and cellular membranes. We first demonstrate our Top-Down MALDI-MS/MS approach on a Photosystem II preparation, analyzing target protein masses between 2.5 and 9 kDa with high accuracy and sensitivity. Then we apply our technique to purify and sequence the mycobacterial ATP synthase *c* subunit, the molecular target of the antibiotic drug bedaquiline. We show that our approach can be used to directly track and pinpoint single amino acid mutations that lead to antibiotic resistance in only 4 h. While not applicable as a high-throughput pipeline, our MALDI-MS/MS and ISD-based approach can identify and provide valuable sequence information on small membrane proteins, which are inaccessible to conventional Bottom-Up techniques. We show that our approach can be used to unambiguously identify single-point mutations leading to antibiotic resistance in mycobacteria.

## Introduction

Bottom-Up LC-MS/MS-based proteomics analyses have become a routine tool for identification and sequence analyses of proteins and allow quantitative studies of thousands of proteins in parallel, tracking protein abundances, turnover, and post-translational modifications.^1−3^ However, in most of these studies, membrane proteins are largely under-represented. Membrane proteins lack proteolytic cleavage sites for commonly used proteases, and the (few) peptides often do not elute efficiently from the reversed-phase columns or display only limited ionization efficiencies in electrospray ionization. These issues are exacerbated for small membrane proteins below 10 kDa where sequences are often poorly annotated and many preparative protocols include filter-based purification steps (to remove small molecular weight contaminants) that also deplete small proteins.^4^ In addition, small membrane proteins have a low number of soluble segments or loops that could yield tryptic peptides, even though sometimes even a single polar loop can provide high-quality peptides sufficient for identification.^5,6^

Different strategies have been developed to address these challenges. Optimized solubilization and digestion protocols drastically increase the yield of membrane proteins,^7,8^ and alternative proteases such as chymotrypsin,^9^ pepsin^10^ or elastase^11^ can generate proteolytic peptides also from transmembrane (TM) segments lacking amino acids with basic side chains and can provide valuable identification and sequence information data, even though quantitation is compromised due to missed cleavages. Chromatographic separation using C4- or C8-based reversed-phase materials, elevated temperatures, and 2-propanol as a solvent as well as polymeric column bed materials, such as PLRP/S, have additionally improved the yield in membrane proteins.^12−14^ Recent developments and methodological improvements in Top-Down mass spectrometry now enable researchers to ionize relatively large protein complexes^15,16^ and obtain meaningful sequence information, also by combining different fragmentation strategies.^17−19^ However, most Top-Down studies still focus on soluble proteins because of chromatography and electrospray ionization.^13^

Small membrane proteins remain largely unexplored, and we and other laboratories recently identified novel, small subunits in supposedly well-studied membrane protein complexes. For example, we identified and confirmed novel small subunits in photosynthetic complex I using alternative proteases^20^ and studied their function in the complex.^21^ In other projects where alternative proteases did not yield sufficient high quality peptides, we were able to characterize these subunits using organic solvent- and solid phase-based extraction methods to purify the samples and MALDI-TOF/TOF mass spectrometers optimized for high *m*/*z* detection to directly sequence the polypeptides without previous proteolytic digestion using MALDI-ISD or MALDI-MS/MS analyses. MALDI-ISD (MALDI In-Source Decay)^22^ is a pseudo-MS/MS technique that typically provides a very high sequence coverage for proteins <20 kDa.^23−25^ However, MALDI-ISD does not allow precursor ion selection as the fragmentation occurs in the MALDI plume during ionization in the source by charge-transfer reactions and radical-driven reactions, primarily leading to c- and z+2-ions.^26,27^

For digest-free, Top-Down analyses of multimeric protein complexes, MALDI-MS/MS allows isolation and fragmentation of specific precursors. For example, we found and identified new subunits in *cbb*_*3*_ cytochrome *c* oxidase of *Pseudomonas stutzeri*,^28^ in the *bd* oxidase of *Escherichia coli*,^29^ and in photosystem II (PSII) assembly intermediates from *Thermosynechococcus elongatus*.^30^ Here we present this workflow in more detail and showcase the sequencing power on several target proteins. We then apply our technique to the *c* subunit of the mycobacterial ATP synthase (∼8.5 kDa), which is the target of the 2012 FDA-approved antimycobacterial drug bedaquiline used to treat multidrug- and extensively drug-resistant tuberculosis.^31,32^ In some cases, single point-mutations in the *c* subunit are responsible for antibiotic drug resistance, and direct, PCR-free sequence determination from bacterial lysate would allow immediate decision if bedaquiline or other drugs should be used for antibiotic treatment.^31,33−37^ We show that our approach allows direct identification and sequence analysis of wild type and variants, including positional tracking of 1 Da single point mutations leading to antibiotic resistance, in a workflow taking less than 4 h directly from microgram amounts of bacterial cells.

## Materials and Methods

### Rapiflex Method and Calibration

The rapifleX MALDI-TOF/TOF instrument (Bruker) was used with a high mass acquisition method with dedicated high mass calibrants. Calibrant proteins: insulin (no. I5500), ubiquitin (no. U6253), and thioredoxin (no. T0910) were ordered from Sigma-Aldrich and prepared as stock solutions at 50 pmol/μL in TA30 (70% water, 30% acetonitrile (ACN), and supplemented with 0.1% trifluoroacetic acid (TFA)). Asialofetuin (no. A4781, Sigma) was reduced (dithiothreitol), alkylated (iodoacetamide), and digested using trypsin (Promega) according to standard vendor protocols. Each protein was mixed with Super-DHB (sDHB, Bruker) matrix solution (50 mg/mL) and 1 μL directly spotted on a ground steel MALDI target plate (Bruker).

### Protein Preparation

#### Photosystem II

The protein production and purification were conducted as described previously.^30^ The target protein was purified and desalted using Isolute C18 SPE cartridges (Biotage, Sweden). The columns were first washed and equilibrated, the sample diluted in 0,1% TFA and loaded onto the column. After washing with 2 mL 0.1% TFA the protein was eluted with 500 μL 80% ACN, 20% water. The organic fraction was lyophilized in a vacuum concentrator (Eppendorf, Germany), reconstituted in 0.1% TFA and mixed in a 1:1 ratio with HCCA matrix solution (HCCA (alpha-cyano-4-hydroxycinnamic acid) saturated in TA50 (50% ACN, 50% water and supplemented with 0.1% TFA). Subsequently 1 μL aliquots of the mixture were deposited on a ground steel MALDI target and allowed to dry and crystallize at ambient conditions.

MS and MS/MS spectra were acquired on the rapifleX MALDI-TOF/TOF in positive-ion mode. The Compass 2.0 (Bruker) software suite was used for spectra acquisition and processing (baseline subtraction, smoothing, peak picking) and BioTools 3.2 (Bruker) for manual spectrum interpretation, *de novo* sequencing and peak annotation (using a *T. elongatus* database downloaded from Uniprot 4/2019).

#### ATP Synthase *c* Subunits

*M. phlei* cells were grown as described previously (in brief, for 4 days at 37 °C in 10 mL of 7H9 medium)^38^ and harvested by centrifugation at 15000*g* at 4 °C. The cells were resuspended in 10 mM Tris/HCl pH 8.0 to a concentration of 50 mg cells/mL. For sensitivity tests, the cell concentration was diluted using resuspension buffer to the indicated values in each extraction. In each experiment, a total volume of 50 μL cell suspension was used per extraction and mixed in an Eppendorf cup with 500 μL of a 1:1 (v/v) mixture of CHCl_3_/MeOH and incubated at 30 °C for 1.5 h with gentle agitation. Next, the samples were centrifuged for 10 min at 15000*g* to pellet precipitated material. After pellet removal, phase separation was induced by the addition of 100 μL of resuspension buffer to the supernatant. After vortexing for 60 s followed by centrifugation at 13000*g* for 2 min to clear the phase separation, the lower (organic) phase was collected and dried in a SpeedVac (no heating). The dried protein pellets were stored at −20 °C and later used for MS measurements.

*M. phlei* WT and D32N *c*-rings were overexpressed using a pT7–7 vector in *E. coli* BL21 (DE3). Overexpression was performed for 20 h at 37 °C using autoinduction medium^39^ supplemented with 200 μg/mL ampicillin. Cells were pelleted and resuspended in membrane buffer (20 mM Tris/HCl pH 8.0, 50 mM KCl) and lysed using a Cell Disruptor device (Constant Systems Ltd.) in the presence of 1 mM Pefablock and DNase, and membranes were collected by centrifugation (235.000*g*, 1 h, 4 °C). Membrane pellets were resuspended in membrane buffer to 10 mg/mL and solubilized for 15 min at 65 °C in the presence of 2% sodium lauryl sarcosin (LS) and 5 mM ethylenediaminetetraacetic acid (EDTA). Precipitated material was removed by centrifugation (235.000*g*, 1 h, 4 °C), 72% (NH_4_)_2_SO_4_ was added to the supernatant, and the sample was incubated for 30 min at room temperature, followed by a centrifugation and filtration. The clarified sample was dialyzed (20 mM Tris/HCl pH 8.0) overnight and submitted to a Q-sepharose column (wash buffer A: 20 mM Tris/HCl pH 8, 0.2% dodecyldimethylaminoxid (LDAO), wash buffer B: 20 mM Tris/HCl pH 8, 0.2% LDAO, elution buffer: 20 mM Tris/HCl pH 8, 0.2% LDAO, 1 M NaCl). The eluted sample was desalted into 20 mM Tris/HCl pH 8, 0.2% LDAO and concentrated using centrifugal concentrators (PES, 30k MWCO, Vivaspin) before applying it onto a MonoQ column (Cytiva). The column was washed with 20 mM Tris/HCl pH, 0,2% LDAO and the c-ring eluted in a gradient from 0 to 100% NaCl. The purified c-ring sample was concentrated using centrifugal concentrators (PES, 30k MWCO, Vivaspin) and stored at 4 °C for further usage.

Organic extraction of c-subunits from purified c-ring samples was performed by mixing 1 μL c-ring sample with 100 μL of (1:1, v/v) CHCl_3_/MeOH. Phase separation was initiated by addition of 20 μL 10 mM Tris/HCl pH 8.0 with the c subunit extracted into the organic solvent. The organic phase was evaporated, and the dry pellet was stored at −20 °C until MS analysis.

For the tissue lysate mixing experiments, mouse lung tissue was obtained from other experiments conducted in the MPI for Brain Research according to standard protocols. Lung tissue was homogenized using lysis/resuspension buffer and mechanical shearing force and mixed with mycobacterial lysate in indicated ratios. The samples were then extracted using the protocol described above for purification of c-subunits.

For low resolution screening experiments, 1 μL was mixed in a 1:1 ratio with 2,5-dihydroxybenzoic acid (DHB) matrix (30 mg/mL in TA50, Bruker) and deposited on a ground steel MALDI target and allowed to dry and crystallize at ambient conditions. MS data were acquired on a Autoflex III Smartbeam MALDI-TOF/TOF mass spectrometer (Bruker), operated in linear positive ion mode using the default method for a mass range of 5000–20000 *m*/*z*.

For MS/MS analysis, the dried protein extract was resuspended in TA50, and aliquots were spotted onto big anchor targets. After incubation for 1 min, the sample was removed from the target and the dried spots were rinsed multiple times with 0.1% TFA. Finally, 0.5 μL of matrix solution (25 mg/mL sDHB in 50% ACN, 50% water and 0.1% TFA) was added and allowed to dry and crystallize at ambient conditions. MS spectra as well as ISD and MS/MS fragment ion spectra were acquired on the rapifleX TOF/TOF mass spectrometer and analyzed manually with flexAnalysis and Biotools 3.2.

Mixture data were processed using the MALDIquant and MALDIquantForeign packages^40^ in R. Briefly, all spectra were preprocessed using smoothing and baseline removal followed by PQN normalization, peak picking, and alignment.

## Results and Discussion

### High Mass MALDI-MS/MS Calibration and System Performance

**Figure 1:**
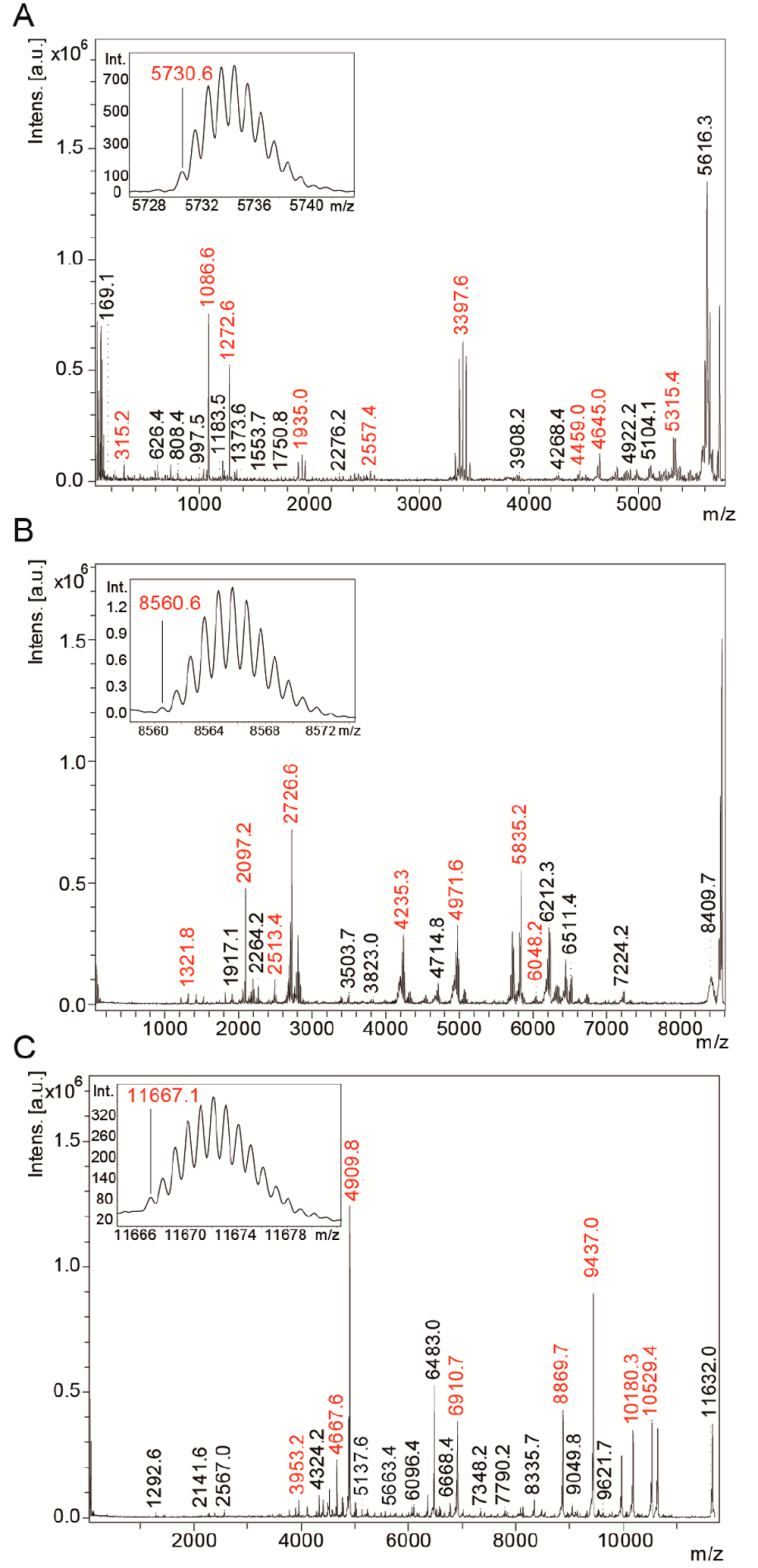
MALDI-MS/MS spectra for high mass calibration. Inset: resolved ion patterns with monoisotopic peaks assigned. (A) Insulin (*m*/*z* 5730.6), (B) Ubiquitin (*m*/*z* 8560.62), (C) Thioredoxin (*m*/*z* 11667.06).

First, we optimized and benchmarked our reflector voltages using a set of well-characterized reference peptides and proteins as outlined in the Materials and Methods, which are now available on all rapifleX instruments. We chose a mixture of three proteins (insulin, ubiquitin, thioredoxin) and used them as calibration standards for our MS and MS/MS measurements (Figure 1). These standards enabled us to optimize resolution, mass accuracy, and sensitivity over the full mass range up to 11.7 kDa (Figure 1C). We then tested our calibration and fragmentation efficiency on five glycopeptides derived from asialofetuin (Supplemental Figure 1), with precursor masses ranging from 5 to 7.1 kDa, obtaining extensive sequence tags and comprehensive sequence coverage for the three peptides (Supplemental Table 1). For the N-linked asparagine glycans, we made use of the MALDI fragmentation pattern to determine and identify the peptide sequence and glycans (Supplemental Figure 1). Based on these observations, we then set out to test how well our methodological approach works on polypeptides purified from membrane protein complexes.

To obtain high-quality MALDI-MS/MS data, we applied and tested different organic solvent- and solid phase-based extraction methods for each sample. In general, the target membrane proteins were solubilized in non-PEGylated detergents with low critical micelle concentration to facilitate removal, as outlined in the Materials and Methods. Each preparation included a final purification step using organic solvent; this approach led to a significant improvement in matrix crystallization and a decrease of salt adduct peaks in the spectra.

### MALDI-MS/MS Based Sequencing of Small PSII Subunits

**Figure 2:**
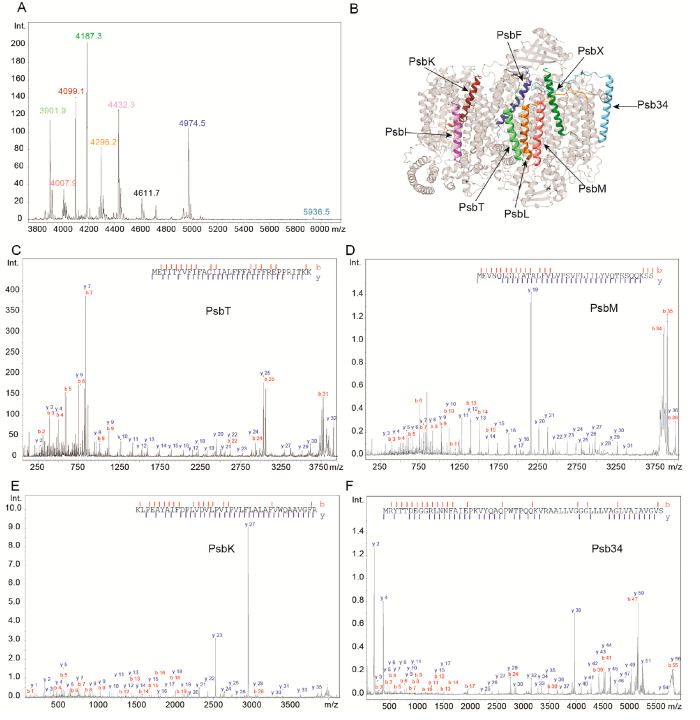
(A) Overview MALDI-MS spectrum of the identified small subunits of Photosystem II (PSII) after purification with C_18_ SPE and spotting with sDHB. (B) Structure of the PSII with highlighted small subunits (PDB: 7NHP). Colors refer to peak labels in panel A. (C–F) MALDI-MS/MS spectra of PSII small subunits with assigned b- (red) and y-ion (blue) series and their respective sequence coverages as indicated by dashes in the sequence: PsbT (theoretical: *m*/*z* 3901.1, 93.8% sequence coverage, including N-terminal formylation), PsbM (theoretical: *m*/*z* 4007.18, 100% coverage, including N-terminal formylation), PsbK (theoretical: *m*/*z* 4098.32, 97.3% coverage), and Psb34 (theoretical: *m*/*z* 5936.24, 76.8% sequence coverage).

We applied our approach to a complex sample, a preparation of a PSII assembly intermediate from *T. elongatus*, with 12 known small subunits, all in the range of *m*/*z* 3000–8000, which has been well studied using conventional Bottom-Up and ESI-/MALDI-based Top-Down methods.^41,42^ We purified the polypeptide chains using C18 SPE extractions, redissolved the dried extracts, and spotted the preparations with sDHB and HCCA on ground steel targets (Materials and Methods). Figure 2A shows an overview mass spectrum of such a preparation, with numerous small subunits clearly visible in a relatively small *m*/*z* range, but mostly without overlapping isotopic envelopes. Because of the relatively large isolation windows in a TOF/TOF experiment, this is a prerequisite for specific isolation of each of the polypeptide chains. Panels 2C–F of Figure 2 show MS/MS spectra of the indicated peaks in Figure 2A and in the structure in Figure 2B, with the fragment patterns annotated based on the sequences of the respective polypeptides. Notably, sequence information was easily sufficient to unambiguously identify each subunit. However, the sequence coverage also varied depending on polypeptide sequences and charge distributions. Subunits PsbK (97.3%), PsbM (100%), PsbT (93.8%), and PsbX (97.5%) showed excellent sequence coverage and signal-to-noise ratios (Figure 2), but PsbL yielded only three sequence tags with 45.9% sequence coverage, with subunits PsbI (50.0%) and PsbF (65.9%) displaying similar results (Supplemental Figure 2). We also want to point out that the signal intensities for the different subunits varied by 2 orders of magnitude between the highest (PsbX) and lowest (Psb34) peak. The PSII assembly factor Psb34, even though it is the protein with the lowest intensity, still yielded a sequence coverage of 76.8% (Figure 2D). Taken together, our digest-free, Top-Down sequencing method enables selective isolation and identification of individual small membrane protein subunits up to 5.7 kDa in the intermediate PSII complex.

### Optimized Extraction and Detection of Mycobacterial ATP Synthase *c* Subunits

We then tested our approach on the *c* subunit of a nonpathogenic model ATP synthase from *Mycobacterium phlei*, which matches 100% of the core *c* subunit amino acid sequence of *Mycobacterium tuberculosis*.^38^ The ATP synthase *c* subunit is an integral membrane protein of ∼8.5 kDa forming a two transmembrane-helix hairpin structure with a short, polar linker loop. The *c* subunits are well-known to oligomerize into homo-oligomeric complexes, so-called *c*-rings, forming an hourglass shaped cylinder with a central pore.^43^ The *c*-rings are the central, membrane-embedded rotor component in ATP synthases and are responsible for the reversible bind/release of protons or sometimes Na^+^ for ion translocation across the membrane, which drives ATP synthesis. The outer surface of the rotor *c*-ring comprises the access site for ions and is at the same time the target of the antituberculosis drug bedaquiline. The mode of action of bedaquiline has been described to bind at the mycobacteria’s *c*-ring ion binding site and block its rotational mechanism leading to a complete standstill of the ATP synthase motor function in this pathogen, killing *M. tuberculosis* bacteria due to a lack of cellular ATP supply.^31,38,44^ While bedaquiline binds highly specifically to ATP synthases in *Mycobacteria*,^38^ both in vitro selected and the recent appearance of single point mutations have shown to diminish the drug’s high activity to kill *M. tuberculosis* in multidrug resistant cases and last line treatment approaches.^31,34−37^ Notably, in all these cases, the structural environment around the binding pocket and an essential ion binding glutamate E61 were identified to be affected in these bedaquiline resistant mutants, in line with initial findings when bedaquiline was discovered and point mutations were screened in *M. smegmatis* (e.g., D32V).^31^ In clinical *M. tuberculosis* isolates, showing elevated bedaquiline minimal inhibitory concentrations (MICs), these positions were identified in the *c* subunit: G25S, D28G, D28N, E61D, A63P, and A63V.^34^ So far, its identification requires tedious and time-consuming isolation and antibiotic resistance testing procedures in a higher biosafety level categorized laboratory. A fast, low biosafety demanding, and accurate identification of bedaquiline-resistant mutants would thus provide a highly valuable diagnostic tool to quickly decide which therapy against an emerged multidrug resistant *M. tuberculosis* infection is advisible.

**Figure 3:**
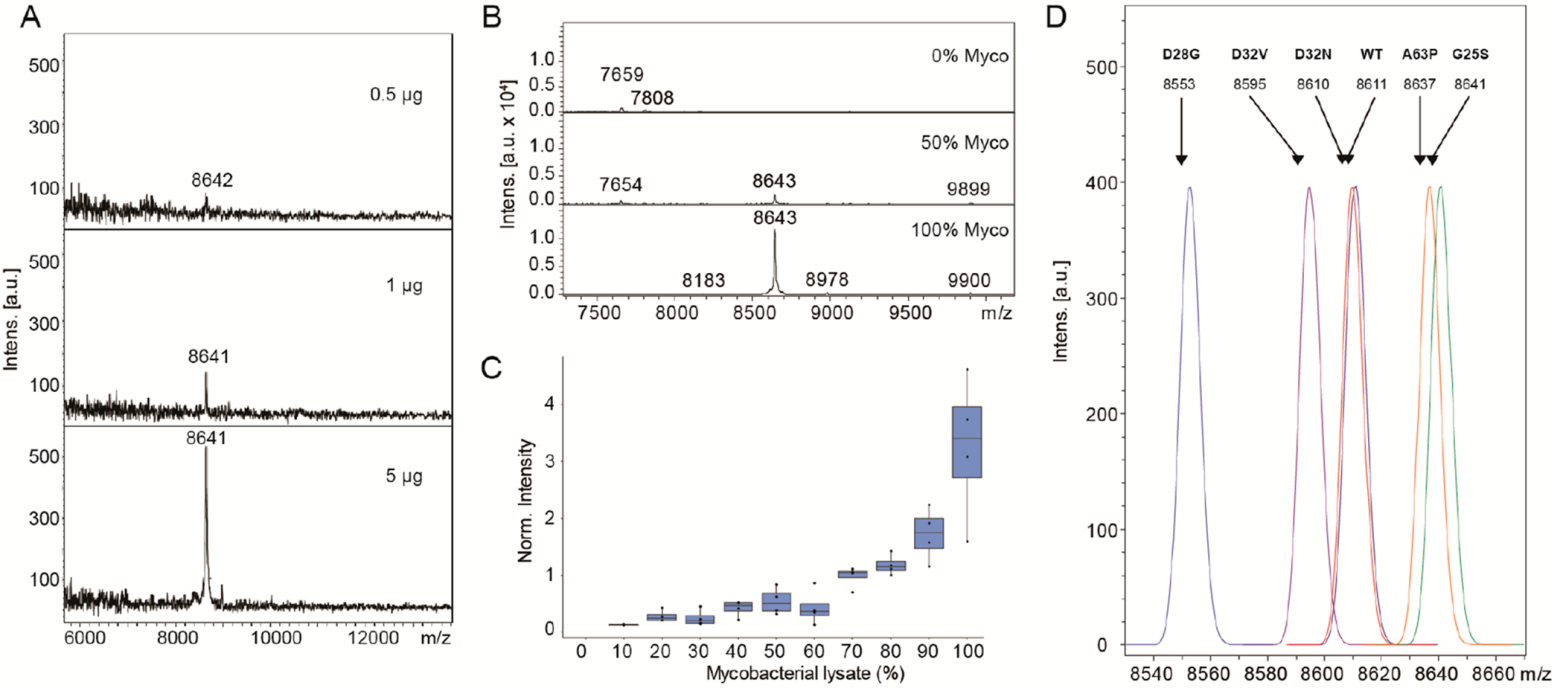
(A) LOD determination for the extraction of *M. phlei* ATP synthase *c*-subunit (N-terminal formylated, theoretical average molecular weight: 8638 Da) from 0.5 to 5 μg of cell lysate. (B) Recovery of *c*-subunit signal from mixtures of the lysate with lung tissue lysate (0, 50, 100% mixing ratios). (C) *c*-Subunit mass intensities from lysate mixtures for *n* = 4 replicates. (D) Simulated peak patterns for *c*-subunit mutations originating from the mycobacterial ATP synthase c-ring in vivo, before extraction.

We thus set out to acquire sequence information directly from bacterial lysate by combination of a simple and low biosafety level requiring organic extraction protocol followed by digest-free, direct MALDI-MS/MS. Although previous studies succeeded with a fast extraction according to Wessel and Fluegge,^45^ the mycobacterial samples, in contrast to for example ATPase *c*-subunits from *Arabidobsis thaliana* thylakoid membranes,^46^ required a prolonged extraction time and harsher conditions for sufficient analyte recovery from the particularly stable and complex mycobacterial cell wall.^47^Figure 3A shows MALDI-MS spectra of different amounts of starting material, ranging from 0.5 to 5 μg extracted lysate. These starting amounts represent a fraction of the material obtainable in a standard biopsy or a bronchial lavage.^48^ Notably, we acquired these initial screening data on a Bruker Autoflex III Smartbeam mass spectrometer, in linear positive ion mode. The resolution in this acquisition mode is comparable to low-resolution mass spectrometers frequently used in hospitals for microbial detection and analysis (e.g., Bruker Biotyper or Shimadzu MALDI 8020). The observed *m*/*z* values were stable across the entire extraction protocol, and even acquisition and summation of high shot numbers and higher laser energies at lower sample amounts (5/6-fold between 0.5 and 5 μg) were easily compensated by near-neighbor calibration. As clinical samples are frequently derived from biopsies or bronchial lavages that can contain lung tissue contaminations from the patients, we mixed our mycobacterial lysates with different ratios of mouse lung tissue prepared separately. Figure 3B shows representative mass spectra for the dilution range of lysate mixtures from 0%–100% of mycobacterial lysate to lung tissue ratios in 10% step increments. The target *c* subunit peak at *m*/*z* 8643 (avg. mass) is clearly detectable until a mixture ratio of 70% of mycobacterial lysate with high confidence, and a weak peak remains detectable until 20% mycobacterial lysate (Figure 3C). No interfering protein peaks were detectable in any of the extracts. However, at higher lung tissue content we found that the overall spectrum and signal quality was significantly reduced, also with elevated background levels.

Having confirmed efficient and sensitive extraction and detection, we then performed the same analysis on ATP synthase *c* subunits containing single point mutations leading to reduced or abolished bedaquiline binding, thus conveying mycobacterial resistance.^31,34^ The point mutations include addition or removal of bulky side chains leading to steric hindrance or reduced hydrophobic interactions and charge removal (e.g., G25S, D28G, or A63P).^34^ While the former mutations can be tracked using MALDI-MS and accurate mass determination even on low resolution instruments (examples see Figure 3D), some point mutations only lead to a mass shift of 1 Da. In addition, the position of the point mutation is essential, as an amino acid exchange in the soluble domains is unlikely to affect bedaquiline binding. We thus require MS/MS spectra for unambiguous validation of the amino acid sequence and the positions of any putative point mutations (e.g., D28N or D32N).

### Sequencing of ATP Synthase *c* Subunits and Tracking of Point Mutantions

**Figure 4:**
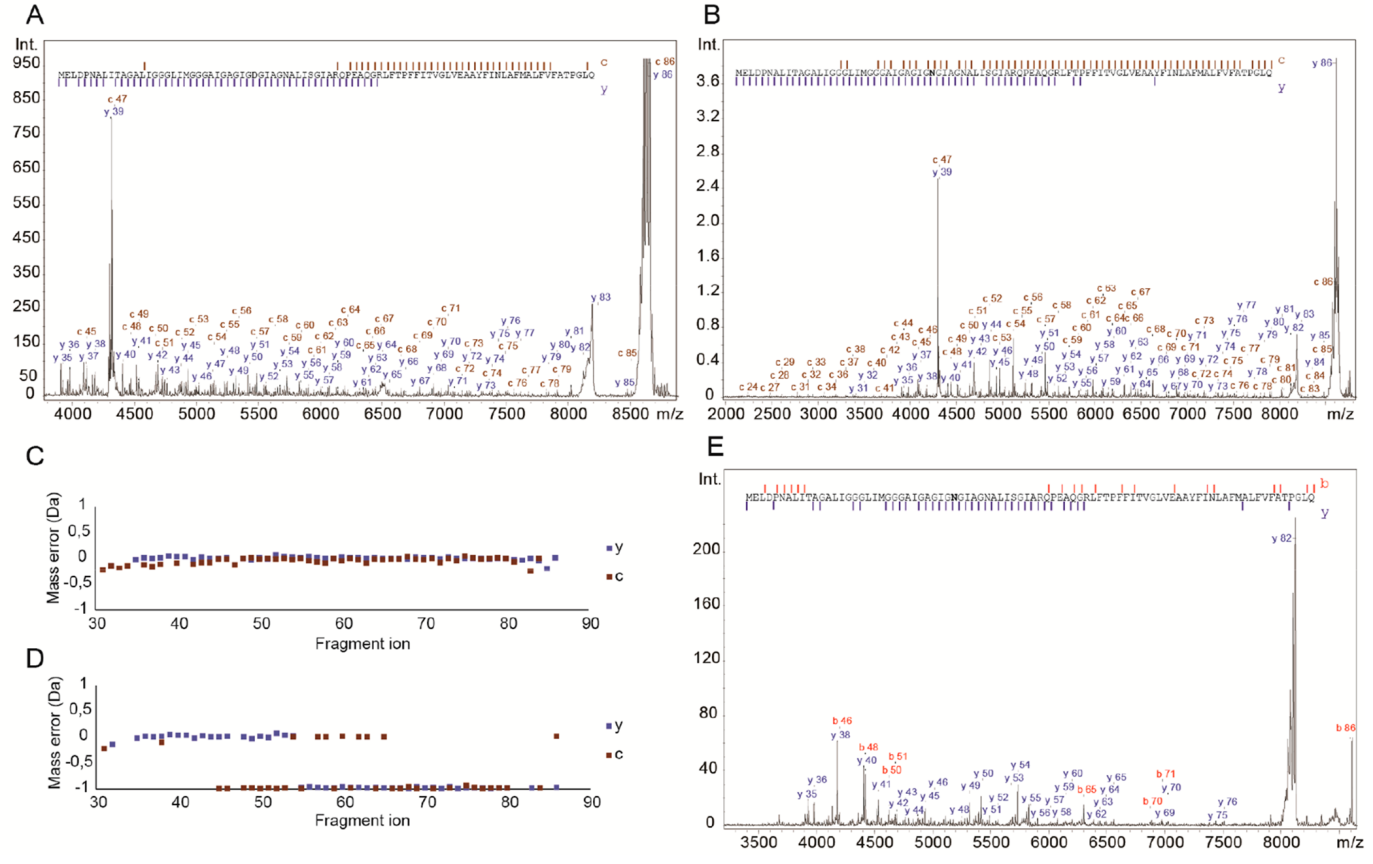
(A) ISD-MS/MS spectrum of wild-type ATP synthase c-subunit (N-teminal formylated, theoretical: *m*/*z* 8633.55, measured precursor: *m*/*z* 8633.5) with assigned c-, y-ion series and the respective sequence coverage (90.7%). (B) ISD-MS/MS spectrum of D32N mutant c-subunit (theoretical: *m*/*z* 8604.57, measured precursor: *m*/*z* 8604.6) with assigned c-,y-ion series and the respective sequence coverage (98.8%). (C) Fragment mass errors across the *m*/*z* range for the D32N sequence mutant (panel B). (D) Fragment mass errors for matching the WT sequence onto the D32N mutant spectrum, clearly indicating 1 Da mass shift. (E) MALDI-MS/MS spectrum of D32N c subunit (theoretical: *m*/*z* 8604.57 *m*/*z*, measured precursor: *m*/*z* 8604.6) with assigned b- and y-ion series and the respective sequence coverage (60.5%), which still covers the relevant mutation sites.

We thus set out to acquire MS/MS spectra of both wild type as well as variant *c* subunits. As our preparations contained no interfering proteins (Figure 3A), we started with the acquisition of MALDI-ISD spectra of WT protein. The respective spectra for WT *c* subunits (Figure 4A) are information-rich and contain extensive and consecutive fragment ions over the whole length of the polypeptide, leading to a sequence coverage of 90.7%. Notably, the c-ion series drops off at position R45 (the c45 ion) and the y-ion series around A44/R45 (y35/36), consistent with the contribution of charged side chains for high quality MALDI-ISD fragment ion spectra.

We then analyzed *c* subunits with the D32N mutation. This mutation completely abolishes bedaquiline binding, as D32 represents a second acidic residue adjacent to E61 and within the proton binding pocket. While it may not be physiologically viable, methodologically, it represents the most challenging variant as the mass difference is just 0.984 Da, and the amino acid exchange is near the middle of the transmembrane segment. We again acquired spectra of a protein preparation purified using organic extraction and obtained excellent signal intensity and signal-to-noise ratio. Notably, the D32N mass shift cannot be discerned in these MS spectra, as the isotopic envelope of an 8700 Da polypeptide is too wide. We thus acquired MALDI-ISD spectra on our D32N preparation, which yielded almost complete sequence coverage (98.8%) across the entire sequence of the polypeptide even exceeding the coverage found for the WT preparation (Figure 4B). This improvement is mainly due to the almost complete c-ion series observed in this spectrum, possibly due to D32N helping to stabilize a charge in this segment. We then investigated the mass accuracy in our ISD-MS/MS spectra, with fragments covering the complete *m*/*z* range up to 8700. We found that the RMS error across the entire range was 0.05 Da, which is easily sufficient to track a putative mass difference of 0.984 Da across the entire *m*/*z* range and thus pinpoint the amino acid position of the exchange (Figure 4C). In fact, the mass accuracy in such a spectrum can be used to assign sequence data and track individual point mutations without a priori knowledge if a point mutation is present in the sample or not. For example, if the D32N MALDI-ISD spectrum was matched to the WT sequence, a mass error of 1 Da occurs at amino acid position 32, and an offset of 1 Da is visible for all consecutive ions (Figure 4D). Notably, the mass difference between the c86 and y86 ions is 1 Da, which leads to the false-positive assignment of the c86 ion with no mass shift in Figure 4D. The method thus can distinguish between WT and single-point-mutation-containing variants that lead to antibiotic resistance.

Our approach could thus be used to directly track if a mycobacterial *c* subunit was prepared from a variant or WT strain sample.^38^ For diagnostic purposes, this technique could therefore comprise a promising alternative to conventional PCR-based analyses which require cultivation and DNA preparation from patient samples. In addition, mixtures of different strains (e.g., a resistant and a nonresistant strain) could make PCR data interpretation extremely challenging, but remain relatively easy to detect on the protein level (two distinct peaks detectable in the MS). Our direct, organic-extraction-based, and digest-free approach could be directly applied to patient samples obtained via bronchial lavages or biopsies. The organic extraction protocol requires only 50+ μg of lysate and takes less than 4 h to complete. Digest-free MALDI-MS/MS analysis then directly provides the data to decide if a mycobacterial strain is treatable by bedaquiline or if other antibiotics need to be administered.

In order to see how well our approach works if a sample has been compromised with lung tissue or other mycobacterial proteins, we then acquired MALDI-MS/MS spectra of the point mutant D32N, selectively isolating and fragmenting the precursor. Figure 4E shows the respective fragment spectrum, which is clearly less information-rich than the ISD spectra. However, the annotated fragment ions still cover 60.5% of the full polypeptide sequence, including the key segment around the cation binding site with amino acid 32. We could thus still unambiguously identify the point mutant D32N in this sample.

## Conclusions

Taken together, our approach allows direct identification and characterization of small, membrane-embedded subunits in larger protein complexes, which are challenging for conventional Bottom-Up proteomics techniques. We complement recently developed Top-Down approaches on ESI-based instruments such as UHMR-configured Orbitraps, which can also provide broad coverage of proteoforms.^13,49^ Our MALDI-based method provides valuable information for the analysis of polypeptides that display poor ionization efficiencies in ESI. In particular membrane-integral proteins are challenging for ESI, such as some ATP synthase *c* subunits from, e.g., *Bacillus pseudofirmus* OF4^50^ and *Pyrococcus furiosus*.^51^ However, *c* subunits from certain species such as *Spirulina platensis* and spinach could be ionized using highly organic and acidified sample solutions.^17,52^ In addition, due to their high hydrophobicity, ATP synthase *c* subunits comprise challenging analytes for reversed-phase chromatography and thus LC-MS. Any attempts to couple our approach to liquid chromatography for analyzing more complex samples will therefore have to address this caveat and will only work for target proteins which do not bind too strongly to reversed-phase chromatography materials.

Notably, MALDI-MS spectra provide singly charged fragment spectra that are relatively easy to interpret and even allow *de novo* sequencing if the signal-to-noise ratio is sufficient.^23^ This was instrumental for our identification of novel subunits in terminal oxidases,^28,29^ as the small subunits were previously not annotated as oxidase subunits and even had conflicting meta-data (e.g., PF05032/CcoM was initially predicted to be an ATP-dependent helicase^28^). The small protein we recently identified in the cytochrome*bd* oxidase in *E. coli*, YnhF, had previously been identified and associated with stress response,^53^ but had not been linked to terminal oxidases.^29^

In particular for targeted approaches on membrane protein complexes purified for structural studies, our approach provides a fast, robust, and sensitive identification of small polypeptides up to 10 kDa. Our setup could potentially provide sequence information for larger proteins as well, but in our experience amino acid distribution and potential predetermined breaking points limit the overall length and quality of the sequence tags with consecutive, usable fragment ions.

In summary, our approach and instrument modification provide a robust and comprehensive method to identify and sequence small membrane proteins, which are largely inaccessible to conventional proteomics approaches. We showed its application for targeted identification and sequence analysis of purified protein complexes for structural studies as well as a diagnostic workflow. We anticipate that it will comprise a valuable tool and complement other approaches for protein sequence analysis and for the quick characterization of multidrug resistant *M. tuberculosis* strains in last-line treatment approaches.

## Supporting Information Available

Calibrant spectra for asialofetuin glycopeptides (Figure S1), MALDI-MS/MS spectra of PSII subunits (Figure S2), Calibrant masses for MALDI-MS/MS (Table S1) (PDF)
