# Supplementary material for: Top-Down Identification and Sequence Analysis of Small Membrane Proteins Using MALDI-MS/MS
Source: J Am Soc Mass Spectrom. 2022 Jun 27;33(7):1293–302. doi: 10.1021/jasms.2c00102 (PMC9264385; doi:10.1021/jasms.2c00102)
Supplement: Supplementary file 1 — js2c00102_si_001.pdf [file js2c00102_si_001.pdf]

Supporting information

Top-Down identification and sequence analysis of small membrane proteins using MALDI-MS/MS

Jakob Meier-Credo[1,2], Laura Preiss[3,4], Imke Wüllenweber[1,2], Anja Resemann[5], Christoph Nordmann[5], Jure Zabret[6], Detlev Suckau[5], Hartmut Michel[7], Marc M. Nowaczyk[6], Thomas Meier[4] and Julian D. Langer*[1,2]

Affiliations

[1] Proteomics, Max Planck Institute of Biophysics, Max-von-Laue-Strasse 3, 60438 Frankfurt am Main, Germany

[2] Proteomics, Max Planck Institute for Brain Research, Max-von-Laue-Strasse 4, 60438 Frankfurt am Main, Germany

[3] Structural Biology, Max Planck Institute of Biophysics, Max-von-Laue-Strasse 3, 60438 Frankfurt am Main, Germany

[4] Department of Life Sciences, Imperial College London, Exhibition Road, SW7 2AZ, London, United Kingdom

[5] Bruker Daltonics GmbH & Co. KG, Fahrenheitstrasse 4, 28359 Bremen, Germany

[6] Department of Plant Biochemistry, Ruhr University Bochum, 44780 Bochum, Germany

[7] Molecular Membrane Biology, Max Planck Institute of Biophysics, Max-von-Laue-Strasse 3, 60438 Frankfurt am Main, Germany

*Correspondence: Julian Langer julian.langer@biophys.mpg.de

Supplement figure 1

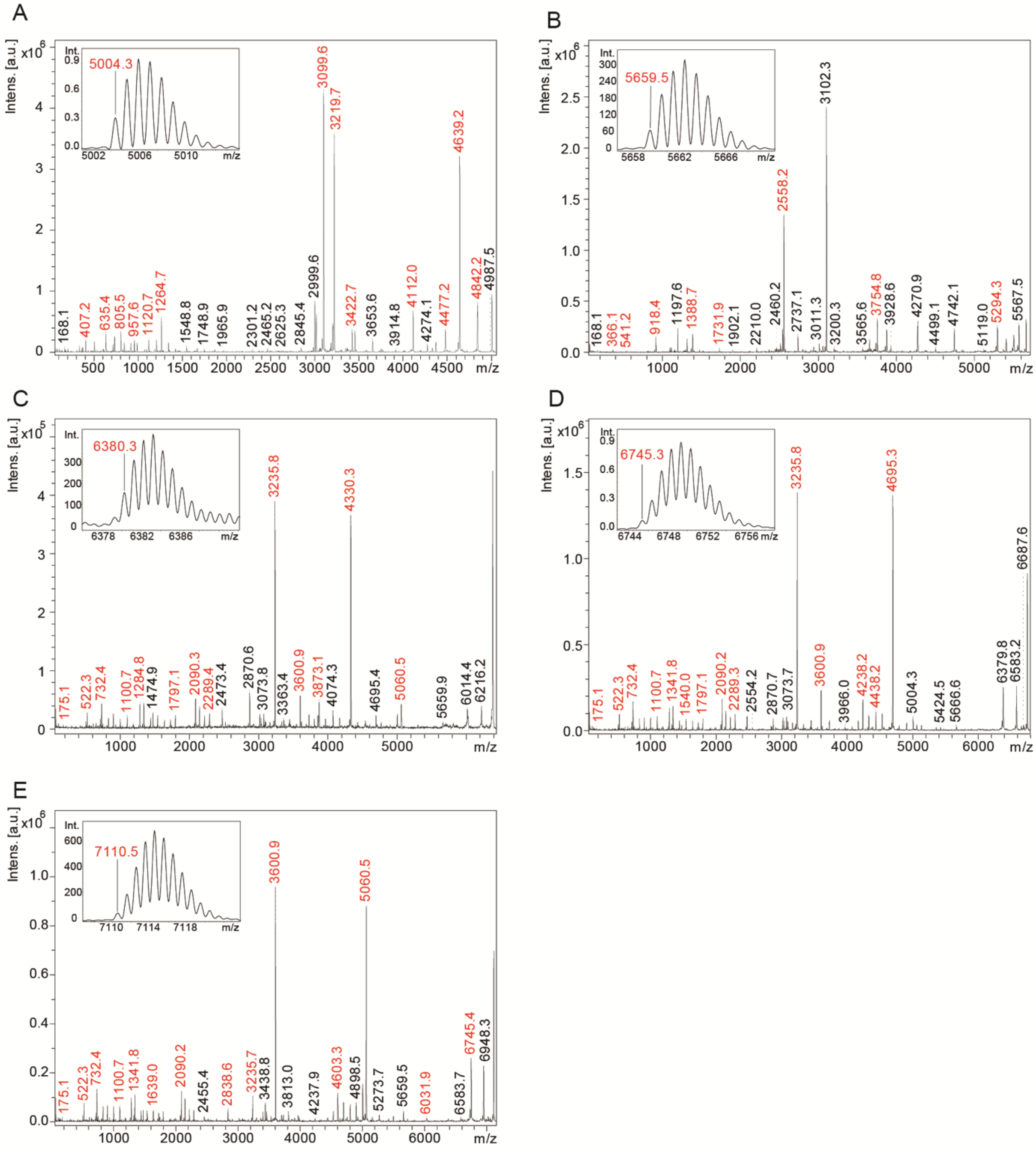

Figure S1: MALDI-MS/MS spectra for tryptic asialofetuin glycopeptides used for calibration. Inserts show the resolved ion patterns with monoisotopic peaks assigned. A) N-glycosylated peptide VVHAVEVALATFNAESNGSYLQLVEISR (theoretical: m/z 5004.29), B) N-glycosylated peptide PTPLANCSVR (theoretical: m/z 5659.48), C) O-glycosylated peptide VTCTLFQTQPVIPQPQPDGAEAEAPSAVPDAAGPTPSAAGPPVASVVVGPSVVAVPLPLHR

with Hex:1 HexNAc:1 (theoretical: m/z 6380.27), D) O-glycosylated peptide VTCTLFQTQPVIPQPQPDGAEAEAPSAVPDAAGPTPSAAGPPVASVVVGPSVVAVPLPLHR with 2 Hex:1 HexNAc:1 (theoretical: m/z 6745.4), E) O-glycosylated peptide VTCTLFQTQPVIPQPQPDGAEAEAPSAVPDAAGPTPSAAGPPVASVVVGPSVVAVPLPLHR with 3 Hex:1 HexNAc:1 (theoretical: m/z 7110.53).

Supplement figure 2

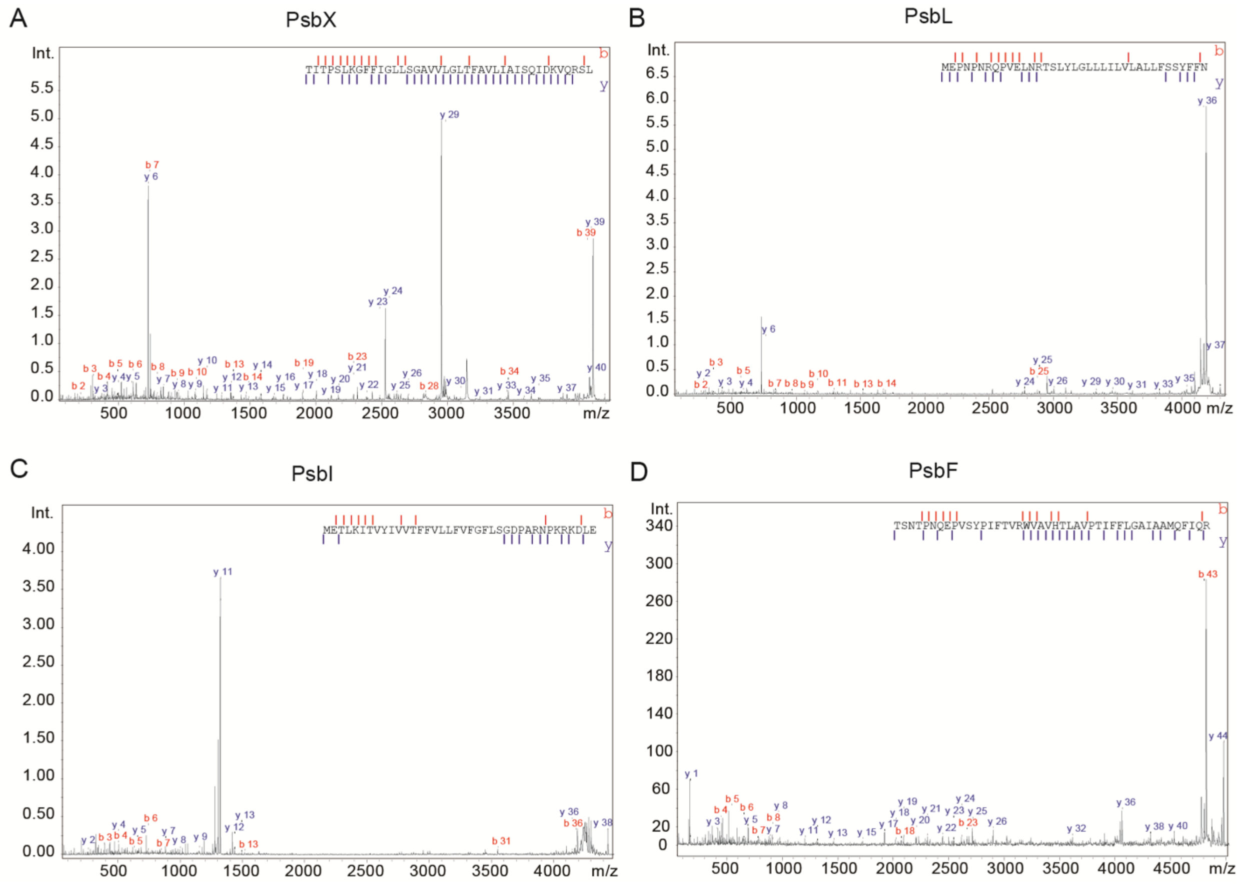

Figure S2: MALDI-MS/MS spectra of PSII small subunits with assigned b- (red) and y- (blue) ion series and their respective sequence coverages as indicated by dashes in the amino acid sequence. A) PsbX (theoretical: m/z 4186.46, 97.5% coverage), B) PsbL (theoretical: m/z 4295.33, 45.9% coverage), C) PsbI (theoretical: m/z 4431.42, 50% coverage, including N-terminal formylation), D) PsbF (theoretical: m/z 4973.62, 65.9 % coverage, including N-terminal acetylation).

Table S1: Calibrant masses for MALDI-MS/MS

Insulin

Ion type	Reference mass
I/L (immonium)	86.10
H (immonium)	110.07
F (immonium)	120.08
Y (immonium)	136.08
y3 (chain 1)	315.20
y9 (chain 1)	1086.57
y11 (chain 1)	1272.64
y17 (chain 1)	1934.98
y23 (chain 1)	2557.29
chain 1 -SH	3363.69
chain 1	3397.67
b19 (chain 1)	4458.98
b21 (chain 1)	4645.04
b26 (chain 1)	5315.37
b29 (chain 1)	5641.56
full protein	5730.61

Ubiquitin

Ion type	Reference mass
P (immonium)	70.07
b3	373.19
a4	492.26
y7	770.5
y8	883.58
y9	1020.64
a10	1091.63
y(-17)10	1116.62
y10	1133.73
y(-17)11	1216.72
y11	1234.77
y12	1321.81
y13	1450.85
y14	1578.94
y15	1707
y16	1820.09

y17	1934.13
y18	2097.19
y19	2212.22
y20	2299.25
y21	2412.34
y22	2513.38
y24	2726.51
b25	2788.46
b(+18)25	2806.475
y(-17)25	2823.53
y25	2841.53
y(-17)37	4235.32
y37	4253.33
y(-17)44	4971.7
y44	4989.7
b51	5720.1
b52	5835.13
b53	5892.15
b54	6048.25
y55	6229.38
b58	6464.44
b60	6741.54
b64	7239.82
full protein	8560.62

Thioredoxin

**Ion type**	**Reference mass**
P (immonium)	70.07
y33	3456.95
y36	3783.15
y38	3953.26
y41	4341.47
y45	4667.63
y47	4909.73
y49	5137.84
y53	5564.1
y58	6062.42
y60	6353.54
y64	6781.73
y65	6910.77

y78	8336.43
y82	8869.66
y88	9437.98
y93	9964.29
y95	10180.37
y98	10529.5
y99	10644.52
b104	11279.85
b106	11464.93
full protein	11667.06

Asialofetuin - N-Glycopeptide

**Ion type**	**Reference mass**
P (immonium)	70.07
y1	175.12
y2	262.15
b3	336.20
y3	375.24
b4	407.24
b5	506.31
b6	635.35
y6	716.43
b8	805.46
y7	844.49
b9	918.54
y8	957.57
b10	989.58
y9	1120.64
y10	1207.67
y11	1264.69
b13	1351.74
b14	1422.77
b15	1551.82
b16	1638.85
b17	1752.89
a19	1868.95
b25	2642.35
full peptide	3016.57
glycan	3099.61
glycan	3219.65
glycan	3422.73

glycan	4111.97
glycan	4477.16
glycan	4639.26
glycan	4842.24
full glycopeptide	5004.29

Asialofetuin - N-Glycopeptide

Ion type	Reference mass
P (immonium)	70.07
y1	175.12
glycan	204.09
glycan	366.14
y4	521.25
glycan	541.27
glycan	803.41
glycan	918.43
glycan	1160.56
glycan	1273.64
glycan	1388.67
glycan	1731.84
glycan	2093.97
glycan	2231.03
glycan	2443.18
glycan	2558.21
glycan	3037.48
glycan	3671.77
glycan	3754.81
glycan	3874.85
glycan	4767.15
glycan	5294.35
glycan	5406.34
glycan	5497.43
full glycopeptide	5659.48

Asialofetuin - O-Glycopeptide (1)

Ion type	Reference mass
P (immonium)	70.07
y1	175.12
y2	312.18
y4	522.32

y6	732.45
y7	831.52
y8	902.56
y9	1001.63
y10	1100.69
y11	1187.73
y12	1284.78
y13	1341.80
y14	1440.87
y15	1539.94
y16	1639.01
y17	1726.04
y18	1797.08
y21	2090.25
y22	2147.27
y23	2218.31
y24	2289.34
y26 +gly	2838.56
y31 +gly	3235.76
y31 +2 gly **	3600.89
y37 +gly	3802.03
y38 +gly	3873.06
y43 +gly	4330.25
y43 +3 gly **	5060.51
full glycopeptide	6380.27

Asialofetuin - O-Glycopeptide (2)

**Ion type**	**Reference mass**
P (immonium)	70.07
y1	175.12
y2	312.18
y4	522.32
y6	732.45
y7	831.52
y8	902.56
y9	1001.63
y10	1100.69
y11	1187.73
y12	1284.78
y13	1341.80
y14	1440.87

y15	1539.94
y16	1639.01
y17	1726.04
y18	1797.08
y21	2090.25
y22	2147.27
y23	2218.31
y24	2289.34
y26 +gly	2838.56
y28 +gly	3036.66
y29 +gly	3093.68
y30 +gly	3164.72
y31 + gly	3235.76
y33 +gly	3447.84
y31 + 2 gly	3600.89
y37 +2 gly	4167.16
y38 +2 gly	4238.20
y40 +2 gly	4438.28
y43 +2 gly	4695.38
y45 +2 gly	4907.46
full glycopeptide	6745.40

Asialofetuin - O-Glycopeptide (3)

Ion type	Reference mass
P (immonium)	70.07
y1	175.12
y2	312.18
y4	522.32
y6	732.45
y7	831.52
y8	902.56
y9	1001.63
y10	1100.69
y13	1341.80
y14	1440.87
y15	1539.94
y16	1639.01
y17	1726.04
y18	1797.08
y21	1993.20
y22	2090.25

y23	2147.27
y24	2218.31
y24	2289.34
y26 +gly	2838.56
y31 +gly	3235.76
y31 + 2 gly	3600.89
y38 +3 gly	4603.33
y40 +3 gly	4803.41
y43 +3 gly	5060.51
y52 +3 gly	6032.02
full peptide +2 gly	6745.40
full glycopeptide	7110.53

**co-isolates from in source fragmentation (e.g. AF4/y58/2gly - 6385 or AF5/y55/3gly - 6389)
